# Home range size, vegetation density, and season influences prey use by coyotes (*Canis latrans*)

**DOI:** 10.1371/journal.pone.0203703

**Published:** 2018-10-10

**Authors:** Jennifer N. Ward, Joseph W. Hinton, Kristina L. Johannsen, Melissa L. Karlin, Karl V. Miller, Michael J. Chamberlain

**Affiliations:** 1 Warnell School of Forestry and Natural Resources, University of Georgia, Athens, Georgia, United States of America; 2 Wildlife Resources Division, Georgia Department of Natural Resources, Social Circle, Georgia, United States of America; 3 Department of Physics and Environmental Sciences, St. Mary’s University, San Antonio, Texas, United States of America; Sichuan University, CHINA

## Abstract

To ensure reproductive success, *Canis* species establish contiguous mosaics of territories in suitable habitats to partition space and defend limiting resources. Consequently, *Canis* species can exert strong effects on prey populations locally because of their year-round maintenance of territories. We assessed prey use by coyotes (*Canis latrans*) by sampling scats from within known territories in southeastern Alabama and the Savannah River area of Georgia and South Carolina. We accounted for the size and habitat composition of coyote home ranges to investigate the influence of space use, vegetation density, and habitat type on coyote diets. Coyote use of prey was influenced by a combination of mean monthly temperature, home range size, vegetation density, and hardwood forests. For example, coyote use of adult white-tailed deer (*Odocoileus virginianus*) was associated with cooler months and smaller home ranges, whereas use of rabbits (*Sylvilagus* spp.) was associated with cooler months, larger home ranges, and less vegetation density. Coyotes in our study relied primarily on nutritionally superior mammalian prey and supplemented their diet with fruit when available, as their use of mammalian prey did not appreciably decrease with increasing use of fruit. We suggest that differential use of prey by coyotes is influenced by habitat heterogeneity within their home ranges, and prey-switching behaviors may stabilize local interactions between coyotes and their food resources to permit stable year-round territories. Given that habitat composition affects coyote prey use, future studies should also incorporate effects of habitat composition on coyote distribution and abundance to further identify coyote influences on prey communities.

## Introduction

Understanding prey selection by predators is a fundamental goal in ecology because it represents an essential ecological process influencing behavior, community structure, and ecosystem productivity. Coyotes (*Canis latrans*), the most widely distributed *Canis* species in North America, exhibit frequency-dependent switching strategies [1,2] under which their moderate body size (9–23 kg; [3]) permits them to use a broad range of mammalian prey that vary from small mammals and lagomorphs to ungulates [4–8]. Although coyotes can exploit a diversity of mammalian prey, they often supplement their summer and fall diet with fruit, such as prickly pear (*Opuntia littoralis*) and American persimmon (*Diospyros virginiana*). In the eastern United States, white-tailed deer (*Odocoileus virginianus*) are thought to be an important food resource for coyotes and there is considerable concern among wildlife managers that coyote predation on deer neonates (i.e., ≤3 mo old) may be significant enough to affect deer populations [9–11]. However, coyote predation of adult deer is believed to be low with most use of adults resulting from scavenging of carcasses made available by human hunters and road kill [12–15].

In the southeastern United States, recent research suggests scavenging by coyotes does not fully explain their use of white-tailed deer and that coyotes are capable of preying on adult deer year-round [7,16]. For example, Hinton et al. [16] suggested that coyotes in eastern North Carolina procured deer through predation rather than scavenging for several reasons. First, they observed a positive correlation between coyote body mass and occurrence of deer in coyote diets, suggesting that body size was an important trait for coyotes to acquire deer through predation since scavenging is opportunistic and should be less affected by body mass. Second, they observed intra- and interspecific segregation of coyote and red wolf (*Canis rufus*) home ranges and suggested that strong site fidelity and defense of territories reduced opportunities for coyotes to scavenge outside their home ranges. Finally, they suggested that energetic returns from carrion did not outweigh increased mortality risks for coyotes when scavenging along roadways and that avian scavengers, such as American crows (*Corvus brachyrhynchos*), black vultures (*Coragyps atratus*), and turkey vultures (*Cathartes aura*), likely reduced opportunities to scavenge roadkill.

Scat analysis is the most commonly used method to determine carnivore diets because it is a noninvasive method with low financial costs that provides a broad picture of food habits [17–19]. However, coyote predation is influenced by intrinsic, social, and environmental factors that are difficult to account for in diet studies that analyze scats. Because coyotes are cooperative breeders with packs spatially segregated on the landscape [20–22], effects of predation on prey species are greatest in areas where territories exist. Accordingly, studies using scat analysis to assess coyote diets should account for packs and defended territories because studies conducted across small study sites (e.g., ≤500 km^2^) may artificially inflate sample sizes and incorporate pseudo-replication in their analyses by treating scats, rather than packs, as sampling units [16,23]. Despite advances in genotyping to discriminate coyote scats from those of other species and to identify unique individuals in populations, pooling scats continues to be a common practice when studying coyote diets [24–27]. By using Global Positioning Satellite (GPS) data from collared coyotes, researchers can spatially target known home ranges of monitored animals to assign scats to meaningful sampling units, such as packs and defended territories, and avoid pseudo-replication and inflated sample sizes. For example, Hinton et al. [16] were able to assess effects of intrinsic (breeder body mass), social (pack size), and environmental (season, agricultural habitat, white-tailed deer abundance) factors on coyote and red wolf diets by collecting scats from known territories and treating packs as sampling units. Therefore, using spatial data to identify home ranges of resident coyotes could improve scat analyses via targeted sampling.

We conducted a broad-scaled assessment of prey use by resident coyotes in two separate populations in southeastern Alabama and the Savannah River area of Georgia and South Carolina. By monitoring approximately 140 GPS-collared coyotes and accounting for residency, we were able to assess the influence of size and habitat composition of home ranges on prey use by coyote packs via scat analysis. In particular, it is difficult to know if occurrences of white-tailed deer in scats were acquired through predation or scavenging. However, diversity of prey use and home range size should provide insights into coyote foraging behaviors. For example, wild pigs (*Sus scrofa*) are a common non-native ungulate in the southeastern United States and, like white-tailed deer, are a popular large game species throughout the region [28,29]. Indeed, coyotes in our study area were reported to be a primary scavenger of wild pig carcasses [30]. If resident coyotes rely on scavenging hunter-killed ungulate carcasses to supplement their diet during winter, we should expect similar seasonal trends of deer and wild pig occurrence in coyote scats. Because scavenging is opportunistic, we should also expect coyote home-range size to be positively correlated with occurrence of deer in their diet. The availability and accessibility of deer carcasses varies spatially and temporally across the landscape and larger home range sizes should increase the probability that resident coyotes locate deer carcasses. By assessing coyote prey use over broad geographic regions and using packs as our sampling units, we sought to better understand how size and habitat composition of home ranges influenced coyote predation on local prey species, such as white-tailed deer.

## Materials and methods

### Study area

**Fig 1:**
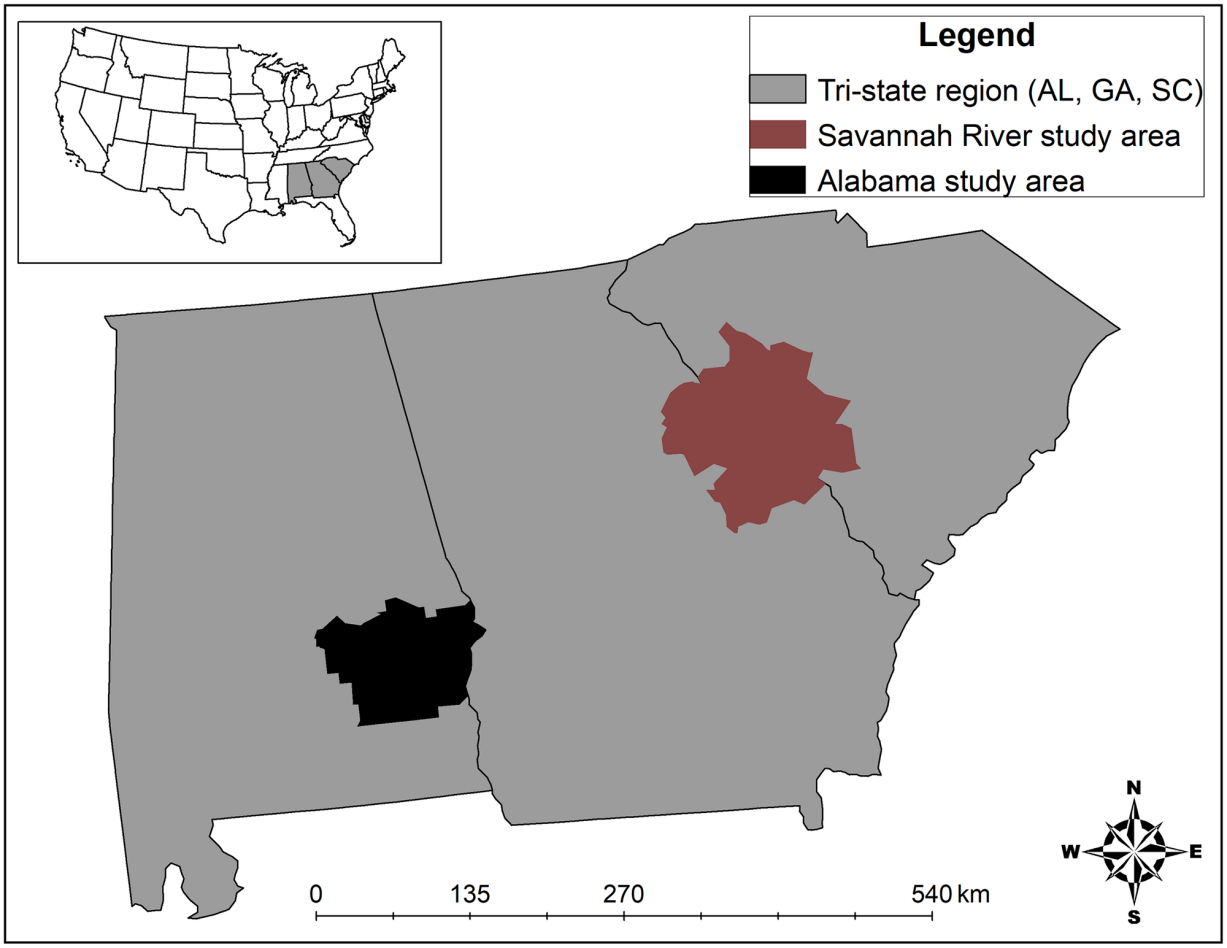
Map of counties (noted as shaded area) in Alabama, Georgia, and South Carolina, USA, where coyotes were trapped during 2015–2016.

The study area encompassed a broad region on private and public lands in southeastern Alabama (Barbour, Macon, and Pike Counties), east-central Georgia (Columbia, Jefferson, Lincoln, McDuffie, and Warren Counties), and western South Carolina (Aiken, Edgefield, McCormick, and Saluda Counties) totaling approximately 16,200 km^2^ (Fig 1). Coyotes captured in Georgia and South Carolina commonly dispersed into each respective study area, and likely represented one population [hereafter the Savannah River area (SRA) population]. Both study areas were situated at the interface of the Piedmont and Southeastern Plains ecoregions and experienced a mild sub-tropical climate with all four seasons. Summers were generally hot and humid with an average high temperature of 20°C, and winters were generally mild with an average low temperature of 1°C [31]. The Piedmont received an average yearly rainfall of 123 cm, whereas the Southeastern Plains received an average of 136 cm [31].

Habitats in the Alabama and SRA contained a mix of early successional, agricultural, forested, and urban habitats. The Piedmont was dominated by loblolly (*Pinus taeda*) and shortleaf (*P*. *echinata*) pine plantations, and successional pine and hardwood forests containing oak (*Quercus* spp.), hickory (*Carya* spp.), sweetgum (*Liquidambar styraciflua*), loblolly pine, and shortleaf pine, and pastures and agricultural fields were also intermittent on the landscape. The Southeastern Plains shared similar characteristics to the Piedmont, consisting of pastures and agricultural fields, pine plantations, and oak-hickory-pine woodlands. Furthermore, the Southeastern Plains contained southern mixed forests with various pines, beech (*Fagus* spp.), sweetgum, southern magnolia (*Magnolia grandiflora*), laurel oak (*Q*. *laurifolia*) and live oak (*Q*. *virginiana*), and floodplains were characterized by oaks, red maple (*Acer rubrum*), sweetgum, American elm (*Ulmus americana*), and areas of cypress (*Taxodium* spp.). Agriculture in these regions included cotton, corn, tobacco, soybeans, and peanuts.

Potential food items for coyotes included rabbits (*Sylvilagus* spp.), wild turkeys (*Meleagris gallopavo*), white-tailed deer, wild pig, eastern woodrats (*Neotoma floridana*), hispid cotton rats (*Sigmodon hispidus*), mice (*Peromyscus* spp.), shrews (*Blarina* spp., *Sorex* spp.), voles (*Microtus* spp.), armadillos (*Dasypus novemcinctus*), opossums (*Didelphis virginiana*), squirrels (*Sciurus* spp.), insects, persimmons (*Diospyros virginiana*), blackberry (*Rubus* spp.), wild plums (*Prunus* spp.), pokeweed (*Phytolacca americana*), wild grape (*Vitis* spp.), muscadine (*Vitis rotundifolia*), and black cherry (*Prunus serotina*) [7,14,32]. Other carnivores in competition for these food items included raccoons (*Procyon lotor*), red fox (*Vulpes vulpes*), gray fox (*Urocyon cinereoargenteus*), and bobcat (*Lynx rufus*).

We recognize that coyotes could directly prey on white-tailed deer via take of fawns and adults, or through opportunistic use of carcasses and animals wounded by hunters. Availability of deer in these two populations differed somewhat due to different hunting season dates and timing of deer reproduction (e.g., breeding and parturition dates). Across our study areas, neonate fawns were available to coyotes for eight months (March—October) of the year. Deer hunting occurred throughout both study sites. In Alabama, hunting began on 15 October and ended on 10 February. In Georgia, hunting began on 10 September and ended on 8 January. Finally, in South Carolina, hunting began 15 September and ended 1 January. Consequently, human hunting provided a seasonally available source of deer to coyotes across six months during fall and winter.

### Data collection

We captured coyotes using foothold traps (Victor #3 Softcatch, Woodstream Corporation, Lititz, Pennsylvania, USA) with offset jaws during January—February 2015–2016. Animals were restrained using a catchpole, muzzle, and hobbles. We determined sex and weight, and estimated age by tooth wear [33]. We categorized coyotes ≥2 years old as adults, 1–2 years old as juveniles, and <1-year-old as pups. Prior to release at capture sites, we fitted coyotes with mortality-sensitive G2110E satellite collars (Iridium; Advanced Telemetry Systems, Isanti, Minnesota, USA). Collars were programmed to record animal locations at 4-hour intervals that were transmitted via the Iridium satellite system every three days to an Advance Telemetry Systems website center for access. No endangered or threaten species were involved in our study and all coyote handling procedures were approved by the University of Georgia Institutional Animal Care and Use Committee (A2014 08-025-R2) and adhered to guidelines published by the American Society of Mammalogists [34]. To access lands to trap and collect scat, state agencies granted permission for publicly owned properties while we obtained permission from landowners to collect data from privately owned lands.

Because our goal was to evaluate prey use by resident coyotes, we determined stability of space use using a rarefaction curve for each animal by calculating monthly home ranges [35]. Similar to Hinton et al. [36,37], we identified resident coyotes as animals that resided in an area and showed stable space use for ≥4 months. We then calculated home ranges and core use areas using 95% and 50% fixed kernel density estimates from utilization distributions performed in Geospatial Modelling Environment using the h-plugin smoothing parameter [38] and ArcMap 10.3 [39].

Once we identified resident coyotes after four months of monitoring, we estimated home ranges and began collecting scats along roads and trails within and proximate to core areas of known territories at least once a month during January 2016–December 2016. We collected scats from packs with ≥ 1 GPS collar and ceased collecting scats from packs when they no longer consisted of individuals with working collars. This approach minimized the chance of collecting scats from transients, as residents typically exclude unrelated conspecifics from their territories [16,22]. For example, Dellinger et al. [40] matched 96% of 196 scats to the genotypes of individual red wolves in territories they sampled, showing inclusion of non-pack member scats was low. Nevertheless, we recognize that our sample likely included scats not belonging to resident coyotes maintaining each territory, but we offer that this occurred infrequently.

We placed scats in plastic bags labeled with the date and a unique identification number and then stored them at -20.0°C for future analysis. We dried scats in an oven at 85°C for 48 h and then recorded dry weights. We then bagged individual scats in nylon stockings with waterproof labels and soaked them in water for 24 h prior to washing. We washed scats in a washing machine twice on the regular cycle with detergent. This separated hair, bone, and other undigested food items from fecal material. We subsequently dried scats in a drying oven at 50°C for 48 h to destroy any zoonotic parasites prior to examining scat contents. To identify prey remains in scat, we visually analyzed each scat for prey items, which we assigned to one of six categories: white-tailed deer, rabbits, small mammals (mice, rats, shrews, and voles), wild pig, plants, and other food items (birds, insects, opossum, raccoon, armadillo, cattle, reptiles and anthropogenic trash). As needed, we examined food items microscopically or compared them to reference collections for identification [41]. To further assess the use of deer, we differentiated adult deer hair in scats from fawns. We measured hair widths using an eyepiece reticule with a microscope and categorized hairs ≤ 80 micrometers as fawns and hairs >80 micrometers as adults [42].

### Statistical analyses

We recorded frequency of occurrence (FO) of each prey category for each scat. When an item constituted ≤ 1% of a single scat it was treated as a trace item and excluded from analyses [19,43]. Because FO is known to overestimate importance of small prey and underestimate importance of large prey [19,44], we estimated percent volume (to the nearest 5%) of each prey item in scats via visual examination and converted percent volume diet estimates to percent biomass to calculate relative total biomass (RTB) of prey in scats. We then used RTB from scats to calculated mean monthly biomass (MMB) ingested by coyote packs for each prey category according to the linear regression model of Weaver [44]. The model converts dry mass of undigested remains of prey in scats into biomass ingested and provides the relative total weight of each type of prey consumed based on the number of scats sampled. Mean body mass estimates for species were necessary to use Weaver’s [44] equation and, because only two prey categories (white-tailed deer and wild pig) consisted of a single species, we used the mean body mass of a common species in each prey category comprising >1 species that was frequently reported as a prey item in coyote diet studies. We used the mean body mass of hispid cotton rats (*Sigmodon hispidus*, 0.1 kg) [45] to calculate RTB of small mammals consumed by coyotes. For rabbits, we used the mean body mass of eastern cottontail rabbits (*Sylvilagus floridanus*, 1.2 kg) [46] to calculate RTB of rabbits consumed. To calculate RTB of fruit consumed by coyotes, we used the mean mass of persimmon fruit (0.05 kg) [47]. Body mass of white-tailed deer in our study ranged between 30–100 kg [48,49]. Body mass of female deer (does) ranged between 30–50 kg, whereas males (bucks) ranged between 40–100 kg. Fawns typically weigh approximately 2 kg at birth during spring and summer and increase in weight until they achieve adult-like sizes in during fall and winter [48,49]. To calculate RTB of deer consumed by coyotes, we used a mean body mass of 35 kg to account for the considerable differences in mass observed for fawns, sub-adults, and adult deer. Because we could distinguish fawn hair from sub-adult and adult hair, we used a mean body mass of 10 kg for fawns and 45 kg for adult deer to calculate the differences in RTB of fawn and adults consumed by coyotes. We used a mean body mass of 85 kg for wild pigs because mass of adult wild pigs ranged between 70–100 kg [50]. We did not calculate RTB consumed by coyotes for other food items because the wide variety of prey (e.g., birds, insects, mammals, reptiles, amphibians) made it impractical to estimate a mean body mass to calculate RTB consumed. Finally, we used FO and MMB estimates for coyote packs to analyze the effect of month on coyote food habits using repeated measures analyses of variance (ANOVAs) and Tukey tests for multiple comparisons.

To assess environmental factors influencing coyote prey use, we included data on season, vegetation density, habitats, and predicted prey distribution in our analysis. Because biological seasons are influenced by phenology, which is largely driven by temperature and known to vary spatially, we incorporated mean monthly temperatures to create a continuous variable that better captured seasonal variation than calendar months. To calculate vegetation density, we estimated vegetation biomass in our study areas using the United States Forest Service National Forest Inventory and Analysis (FIA) dataset, which was created by modeling forest biomass as functions of over 60 predictor layers such as digital elevation models (DEM) and the 1992 National Land Cover Dataset (NLCD).

Within coyote home ranges, we accounted for habitat composition and predicted habitat models of primary prey. We estimated predominant types of vegetation cover using a 30-m resolution digital landscape map of vegetative communities developed by the Southeast Gap Analysis Project [51]. We reduced these predominant vegetation communities to seven primary habitat types and calculated the extent of home ranges consisting of these habitats. We assigned forested habitats to one of three categories: hardwood forests, pine forests, and mixed forests that comprised both hardwood and pine species. We combined grassland, savanna, and early successional habitats into an open and early successional habitat category. We combined herbaceous wetlands, forested wetlands, and riparian habitats into a wetland and riparian habitat category. We categorized agricultural crops and pasturelands as agriculture. Finally, we categorized developed open space, low intensity developed, medium intensity developed, and high intensity developed areas as developed habitats.

To create raster maps of predicted prey distribution for coyotes, we used 30-m resolution digital maps of predicted habitat models for primary prey species available to coyotes developed by the Southeast Gap Analysis Project [51]. Using Spatial Analyst in ArcMap 10.3, we merged raster maps of predicted habitat for common rodents to create a raster map of predicted habitat for small mammals. Similarly, we merged raster maps of predicted habitat for common lagomorphs to create a raster map of predicted habitat for lagomorphs. Finally, we used the Southeast Gap Analysis Project’s raster map of predicted habitat for white-tailed deer as our map of predicted habitat for deer.

After classification of habitat types and predicted prey distributions, we evaluated the degree of redundancy among 12 environmental factors (home range size, extent of 7 habitats in home ranges, extent of predicted habitat for 3 prey types in home ranges, and vegetation density) using principal components analysis (PCA; JMP software; SAS institute; S1 Dataset). Ordination techniques, such as PCA, are popular with community and landscape ecologists because they can identify different types of underlying data structure [52–55]. We used PCA to compress our highly dimensional data set into a lower dimensional one to extract the dominant, underlying gradients of variation (principal components). The principal components (PCs) are weighted linear combinations of the original variables ordered according to the amount of variation each PC explained. We used the latent root criterion (PCs with eigenvalues ≥1) as a stopping rule to determine the number of significant PCs to retain and interpret [52]. We then based our interpretation of each PC on those variables with loadings ≥0.40 or ≤-0.40, and placed most emphasis on those with loadings ≥0.60 or ≤-0.60 [52]. We used the variables with the strongest loadings to interpret the ecological meaning of each PC. The PCs were then used to as indicators of landcover complexes and synthetic variables of landcover gradients existing in our study areas, in which prey consumption either increased or decreased with the value of each of the latent environmental variables.

We used FO of each prey category in scats as a binary response variable (1 = present in scat, 0 = absent from scat) in generalized linear mixed models with a logit link using Program R [56] to determine which environmental factors influenced FO of each category observed in scats (S2 Dataset). These factors included mean monthly temperature and ecologically meaningful PCs consisting of habitats and predicted prey distribution. We then included random intercepts for coyote packs, nested within study area, to account for pack variation. Prior to modeling, we rescaled mean monthly temperature values by subtracting the mean and dividing by one standard deviation. We then used Akaike’s information criterion adjusted for small sample sizes (AIC_c_) and used ΔAIC_c_ to select which models best supported factors influencing FO of prey in coyote scats [57].

## Results

We captured and monitored 164 coyotes across Alabama, Georgia, and South Carolina with GPS collars during 2015–2016. We excluded 17 coyotes from space use analyses due to an insufficient number of relocations. Of the remaining 147 coyotes, 60 coyotes (40.8%) were residents and 48 (26.5%) were transients for the entire time they were monitored, whereas 39 (26.5%) coyotes exhibited both residency and transiency. We collected 1,126 scats from 29 territories during January 2016–December 2016. The number of GPS-collared coyotes in each pack ranged between 1–6 individuals. The mean number of packs monitored each month was 13.4 (SD = 4.8) and ranged between 6–22. The mean number of scats collected per pack each month was 6.9 (SD = 1.8). Mean home range size was 13.5 km^2^ (SD = 7) and varied from 5.4 km^2^ to 39.2 km^2^. Mean core area size and standard deviation across populations was 3.2 km^2^ (SD = 1.3) and varied from 1.0 km^2^ to 6.1 km^2^.

Mean vegetation density within coyote home ranges was 67.5 MG/ha (SD = 18.4) and varied between 32.7 and 92.8 MG/ha. Average percentage of predicted small mammal habitat within coyote home ranges was 51.5 (SD = 15.3) and varied between 19.8%–79.0%. Mean predicted lagomorph habitat within home ranges was 35.8 (SD = 10.3) and varied between 15.0%–55.3%. Average percentage of predicted white-tailed deer habitat within home ranges was 61.6 (SD = 8.9) and varied between 32.7%–73.2%.

Mean percentage of agriculture habitats within coyote home ranges was 15.7 (SD = 11.6) and varied between 0.7%–37.7%. Average percentage of developed areas within home ranges was 6.3 (SD = 4.0) and varied between 0.0%–19.7%. Mean percentage of hardwood forests within coyote home ranges was 8.9 (SD = 13.2) and varied between 0.0%–64.1%. Average percentage of pine forests within home ranges was 31.7 (SD = 18.2) and varied between 0.2%–79.2%. Mean percentage of mixed forests within home was 10.6 (SD = 15.2) and varied between 0.0%–64.1%. Average percentage of open and early successional habitats was 17.3 (SD = 4.4) and varied between 6.5%–26.5%. Finally, mean percentage of wetland habitats was 7.2 (SD = 7.1) and varied between 0.5%–23.4%.

The first three principal components (PC1, PC2, and PC3) of our PCA, which explained 53.2%, 17.0%, and 11.9% of the cumulative variation, respectively, were the only PC scores with eigenvalues ≥1 (Table 1). Since the first three axes explain about 82% of the total variance, we deemed the 3-dimensional solution adequate. PC1 allowed us to distinguish the effect of home range size (extent of space use), as it consisted of all positive loadings (Table 1). PC2 allowed us to distinguish areas dominated by low vegetation density, as it was characterized by negative loadings for vegetation density and habitats typically characterized with understory (mixed forests, and developed areas and roads), and positive loadings for habitats with low vegetation structure (wetland and riparian habitats, and agricultural habitats; Table 1). PC3 allowed us to distinguish between pixels characterized by forested and non-forested areas, as it consisted of positive loadings for hardwood forests, and developed areas and roads, and negative loadings for pine forests and vegetation density (Table 1). Collectively, these PC scores indicated that once PC1 accounted for home range size and extent of habitat types, PC2 and PC3 accounted for variation in vegetation density and hardwood forest, respectively. A disadvantage of PCA is that PCs can be difficult to interpret [58]. However, our interpretation of PCs characterizing habitats within coyote home ranges is aligned with results obtained by other studies suggesting that coyote habitat selection is associated with low vegetation and non-forested habitats [36,59–62].

**Table 1:** Eigenvalues, eigenvectors, and factor loadings of environmental factors assessed within home ranges of coyotes in Alabama, Georgia, and South Carolina of the United States.

Environmental factors	Principal component 1		Principal component 2		Principal component 3	
	Eigenvector	Loading	Eigenvector	Loading	Eigenvector	Loading
Home range size	0.39	0.99	0.01	0.02	-0.05	-0.06
Small mammal distribution	0.38	0.97	0.03	0.04	0.06	0.08
Lagomorph distribution	0.37	0.93	0.17	0.24	-0.01	-0.01
White-tailed deer distribution	0.39	0.98	0.01	0.02	0.02	0.03
Vegetation density	0.11	0.27	-0.49	-0.71	-0.38	-0.45
Wetland/riparian habitat	0.11	0.29	0.53	0.76	-0.19	-0.23
Agriculture	0.19	0.47	0.52	0.75	0.17	0.21
Hardwood forests	0.07	0.18	-0.13	-0.18	0.65	0.78
Mixed forests	0.26	0.66	-0.29	-0.41	0.07	0.09
Pine forest	0.26	0.66	-0.04	-0.06	-0.48	-0.57
Open/early successional habitat	0.38	0.95	-0.05	-0.07	-0.05	-0.06
Developed areas/roads	0.27	0.68	-0.27	-0.38	-0.26	0.43
Eigenvalue	6.38		2.04		1.43	
% of total variance	53.16		17.04		11.92	
Description	Home range size		Vegetation density		Hardwood forests	

**Fig 2:**
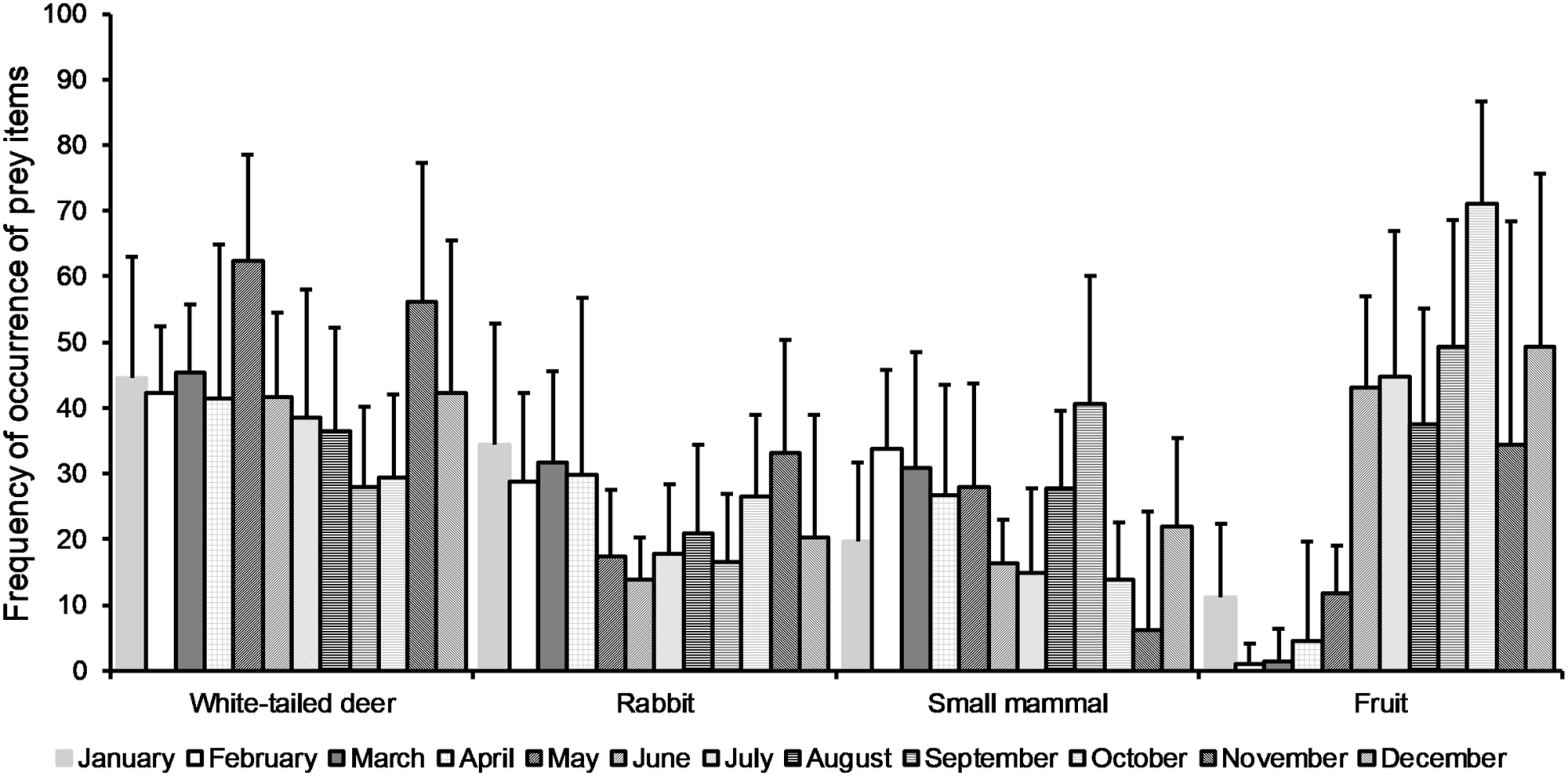
Frequency of occurrence by month of 4 primary prey categories for coyotes in Alabama, Georgia, and South Carolina, USA, 2016–2017. Error bars represent 95% confidence intervals.

**Fig 3:**
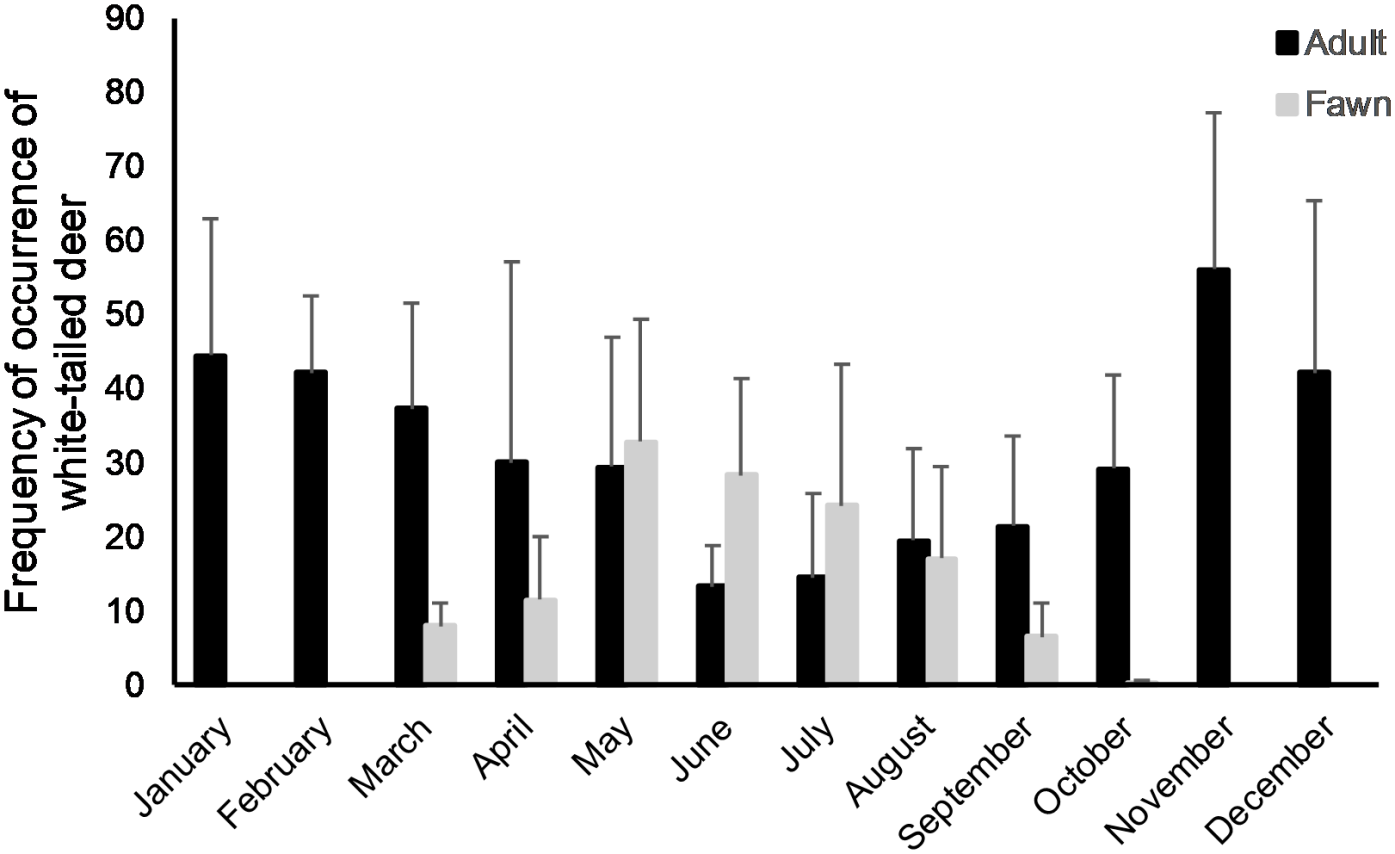
Frequency of occurrence by month of adult and fawn white-tailed deer in coyote scats collected from Alabama, Georgia, and South Carolina, USA, 2016–2017.

Overall, white-tailed deer (40.7%), rabbits (25.1%), small mammals (24.5%), and fruits (27.5%) comprised most prey identified in scats of coyote packs, and since occurrence of wild pig was rare (0.05%), we categorized them with other food items (Table 2). We found that FO differed across months for small mammals (*F*_11,149_ = 2.070, *P* = 0.026) and fruit (*F*_11,149_ = 9.751, *P* ≤ 0.001), but not for rabbits (*F*_11,149_ = 1.340, *P* = 0.208; Fig 2). Although we observed a weak difference in monthly FO of white-tailed deer (*F*_11,149_ = 1.670, *P* = 0.086; Fig 2), monthly difference was more pronounced for adult deer (*F*_11,149_ = 3.558, *P* ≤ 0.001) and fawns (*F*_11,149_ = 4.825, *P* ≤ 0.001; Fig 3). Tukey tests for multiple comparisons of monthly means indicated small mammal consumption was similar among all months. Additionally, Tukey tests revealed FO of fruits peaked during June—December and was lowest during January—May. Consumption of deer peaked during May and was lowest during September but remained similar for all other months. Consumption of adult deer peaked during November—December and was lowest during June—September (Fig 3). Unsurprisingly, FO of fawns was greatest during March—August, as those were the only months fawns were available to coyotes (Fig 3).

**Table 2:** Mean (±SD) frequency of occurrence of primary prey for coyote packs (*n* = 29) in Alabama and the Savannah River area of Georgia and South Carolina, January 2016–January 2017.

	# of scats	White-tailed deer			Rabbit^a^	Small mammal^b^	Fruit^c^	Other^d^
		Total	Adult	Fawn				
Alabama (*n* = 9)	313	36.2±19.0	32.3±19.4	3.8±3.8	17.4±7.3	29.2±14.7	35.1±25.2	14.5±7.6
Savannah River area (*n* = 20)	813	42.8±16.7	28.7±10.5	14.2±10.9	28.6±19.1	22.3±9.9	24.1±20.1	13.3±8.5

^a^Cottontail and swamp rabbit;

^b^Rat, mouse, shrew, and vole species

^c^ Persimmon, wild grape, muscadine, blackberry, dewberry, and pokeweed

^d^Insects (i.e., grasshoppers and beetles), armadillo, livestock, opossum, raccoon, birds, reptiles, and human trash

We found that MMB differed across months for small mammals (*F*_11,149_ = 1.893, *P* = 0.044) and fruit (*F*_11,149_ = 4.445, *P* ≤ 0.001), but not for rabbits (*F*_11,149_ = 1.449, *P* = 0.157). For small mammals, MMB suggested that packs consumed approximately 2.5 kg (SD = 2.0) of small mammals per month (range = 0.1–6.9 kg). For fruit, MMB indicated that packs consumed approximately 7.9 kg (SD = 9.7) of fruit per month (range = 0.0–33.9 kg). The MMB for rabbits suggested that packs consumed approximately 3.4 kg (SD = 2.5) of rabbits per month (range = 1.0–9.5 kg). For white-tailed deer, MMB suggested that packs consumed approximately 12.6 kg (SD = 12.1) of adult deer per month (range = 0.9–29.8 kg). For the 8 months that fawns were available, MMB indicated that coyote packs consumed approximately 2.2 kg (SD = 2.2) of fawns per month (range = 0.0–6.0 kg).

For FO of deer observed in coyote scats, home range size (PC1), mean monthly temperature and, to a lesser extent, vegetation density (PC2) had the strongest effect on model performance (Tables 3 and 4). Coyote use of deer was greatest during cooler months, and consumption of deer was negatively correlated with home-range size and vegetation density (Tables 3 and 4). For FO of adult deer, mean monthly temperature and home range size had the strongest effect on model performance, as coyotes consumed more adult deer during cooler months (late fall—early spring) and coyotes with smaller home-ranges consumed more adult deer than those with larger home-ranges (Tables 3 and 4). For FO of fawns, only mean monthly temperatures had a strong effect on model performance, as fawns were consumed more during warmer months (spring—summer; Tables 3 and 4). For FO of rabbits, home range size and mean monthly temperatures had the strongest effects on model performance, whereas vegetation density exerted a weak effect. Consumption of rabbits occurred more frequently during cooler months, was positively correlated with home-range size and negatively correlated with vegetation density (Tables 3 and 4). Vegetation density and hardwood forest (PC3) exerted weak effects on coyote use of small mammals. Consumption of small mammals was positively correlated with vegetation density and negatively correlated with hardwood forests (Tables 3 and 4). For fruit, mean monthly temperature had the greatest effect on model performance, whereas vegetation density had a weak effect (Tables 3 and 4). Use of fruit by coyotes was greatest during warmer months (late Spring—early Fall) and was positively correlated with vegetation density (Tables 3 and 4).

**Table 3:** Summary of the top 5 generalized linear mixed models used to predict frequency of occurrence of each prey category corresponding to different factors affecting use by coyotes in Alabama, Georgia, and South Carolina during 2016–2017. Shown are differences among Akaike’s Information Criteria for small sample sizes (ΔAIC_c_).

Prey category	Model	*K*	Deviance	ΔAIC_c_	ω
White-tailed deer	Temp^a^+PC1^b^+PC2^c^	5	1496.2	0	0.23
	Temp+PC1	4	1496.7	0.4	0.18
	Temp+PC1+PC2+ PC3^d^	6	1497.1	0.9	0.15
	Temp+PC1+PC3	5	1497.9	1.6	0.10
	Temp+PC2	4	1498.8	2.6	0.06
Adult deer	Temp+PC1	4	1306.2	0	0.46
	Temp+PC1+PC2	5	1307.4	1.2	0.25
	Temp+PC1+PC3	5	1308.2	2.0	0.17
	Temp+PC1+PC2+PC3	6	1309.3	3.1	0.10
	Temp	3	1314.2	8.0	0.01
Fawn	Temp	3	652.3	0	0.26
	Temp+PC2	4	652.6	0.4	0.22
	Temp+PC3	4	653.7	1.5	0.13
	Temp+PC2+PC3	5	653.8	1.5	0.12
	Temp+PC1	4	654.3	2.0	0.10
Rabbit	Temp+PC1+PC2	5	1178.7	0	0.28
	Temp+PC1	4	1178.7	0.1	0.27
	Temp+PC1+ PC3	5	1180.6	1.9	0.11
	Temp+PC1+PC2+PC3	6	1180.6	2.0	0.10
	Temp+PC2	4	1181.7	3.1	0.06
Small mammal	PC2+PC3	4	1279.7	0	0.21
	PC3	3	1280.4	0.7	0.15
	PC1+PC2+PC3	5	1281.2	1.5	0.10
	NULL	2	1281.6	1.9	0.08
	Temp+PC2+PC3	5	1281.7	2.0	0.08
Fruit	Temp+PC2	4	1255.3	0	0.29
	Temp+PC1+PC2	5	1255.5	0.2	0.26
	Temp+PC1+PC2+ PC3	6	1257.2	1.8	0.11
	Temp+PC2+PC3	5	1257.2	1.8	0.11
	Temp	3	1257.6	2.3	0.09

^a^Mean monthly temperature.

^b^Home range size.

^c^Vegetation density.

^d^Hardwood forest.

**Table 4:** Results from top generalized linear mixed models for predicting frequency of occurrence of 6 primary prey corresponding to different environmental factors affecting use by coyote packs in Alabama, Georgia, and South Carolina, 2016. Shown are β coefficients, standard error (SE), 95% confidence intervals (CI), *z*-scores, and *P*-values.

Prey Category	Model Variables	β	SE	95% CI	*z*	*P*
White-tailed Deer	Intercept	-0.457	0.112	-0.689, -0.230	-4.076	<0.001
	Temp^a^	-0.148	0.069	-0.284, -0.013	-2.145	0.032
	PC1^b^	-0.149	0.061	-0.281, -0.034	-2.432	0.015
	PC2^c^	-0.129	0.082	-0.298, 0.034	-1.581	0.114
Adult deer	Intercept	-0.999	0.090	-1.192, -0.828	-11.151	<0.001
	Temp	-0.570	0.072	-0.712, -0.431	-7.964	<0.001
	PC1	-0.151	0.051	-0.262, -0.056	-2.940	0.003
Fawn	Intercept	-2.744	0.244	-3.289, -2.284	-11.248	<0.001
	Temp	1.336	0.178	1.002, 1.714	7.506	<0.001
Rabbit	Intercept	-1.242	0.126	-1.511, -0.995	-9.826	<0.001
	Temp	-0.180	0.079	-0.336, -0.024	-2.274	0.023
	PC1	0.155	0.061	0.036, 0.283	2.547	0.011
	PC2	-0.132	0.093	-0.329, 0.049	-1.425	0.154
Small mammal	Intercept	-1.109	0.097	-1.316, -0.922	-11.399	<0.001
	PC2	0.120	0.071	-0.025, 0.262	1.681	0.093
	PC3^d^	-0.169	0.082	-0.342, -0.007	-2.058	0.039
Fruit	Intercept	-1.120	0.188	-1.537, -0.763	-5.958	<0.001
	Temp	0.482	0.085	0.316, 0.651	5.670	<0.001
	PC2	0.281	0.130	0.017, 0.553	2.157	0.031

^a^Mean monthly temperature.

^b^Home range size.

^c^Vegetation density.

^d^Hardwood forest.

## Discussion

Prey use by coyotes in Alabama and the SRA of Georgia and South Carolina was dominated by white-tailed deer, rabbits, small mammals, and fruit (e.g., persimmon, blackberry, plums, and muscadine). Although these results are consistent with previous studies of coyote prey use in the southeastern United States [7,27,32,63,64], we believe our findings provide important insights because we addressed the problem of pseudo-replication that is common to scat analysis studies [23]. By using coyote packs as our sampling unit, we believe we provided more accurate inferences of coyote prey use than previous studies by accounting for inter-pack variability and correlating prey consumption with size and habitat composition of coyote home ranges. For instance, Schrecengost et al. [14] studied coyote diets proximate to SRA and reported similar use and seasonal changes in mammalian prey and fruit by coyotes. However, they reported that rabbits were not an important food item for coyotes because FO of rabbits only peaked at 31% during February and was <17% during other months. Conversely, our results indicated that rabbits were important prey for coyotes, as monthly FOs for rabbits ranged between 13.8–34.3% and was greatest during January—April (28.7–34.3%), which coincides with coyote breeding and whelping seasons. Our findings differ from Schrecengost et al. [14] because our study area in SRA was considerably larger in size than theirs (10,530 km^2^ vs. 800 km^2^) and we correlated consumption of rabbits to habitat composition of coyote home ranges, finding a negative correlation with vegetation density and use of rabbits. This indicates that rabbit predation by coyotes was greatest in territories with more open habitats and during cooler months of the year. This is an important distinction because 97% of Schrecengost et al.’s [14] study area consisted of forested habitat, whereas our study area comprised 51% of forested habitat. Therefore, we believe our findings demonstrate the importance of accounting for coyote packs and assessing prey use broadly on the landscape to accurately characterize coyote diets.

**Fig 4:**
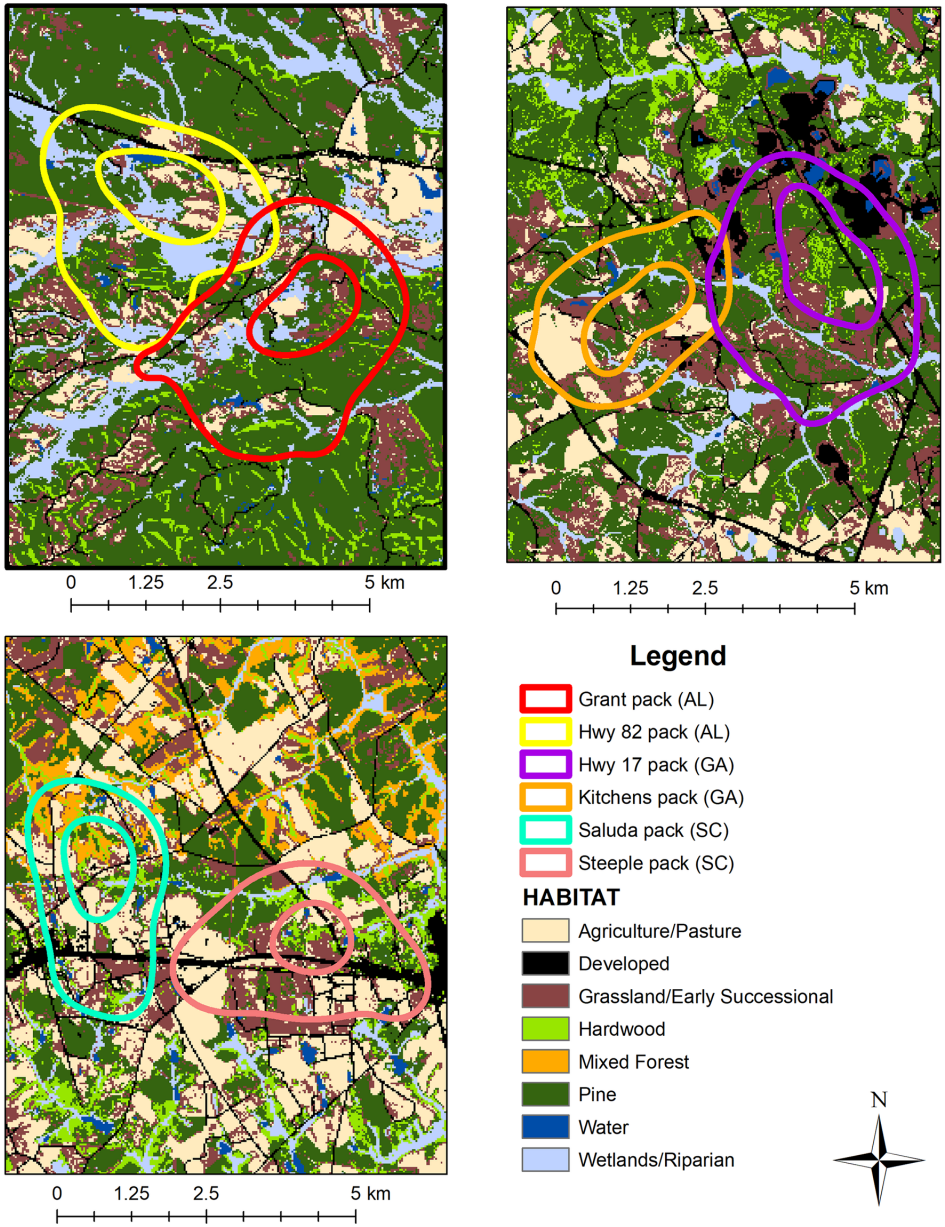
Map showing habitats within 95% kernel density estimated home ranges of 6 GPS-collared coyotes in Alabama, Georgia, and South Carolina 2015–2016.

Territoriality is a behavioral tactic for partitioning space and defending food sources [65–67] that plays a fundamental role in coyote ecology and is rarely accounted for in diet studies (but see [4,16,68,69]). Space use by resident coyotes is constrained by their territorial behavior, as they rarely venture outside their home ranges unless they are dispersing offspring [20,36,70]. Because coyote home ranges contain a finite potential of resources, home ranges reflect a compromise between the cost of energy expenditure to patrol, scent mark, and confront transgressors and the benefits of protecting sufficient resources (e.g., food, dens, refugia) that enabled coyotes to maintain stable breeding territories [71–75]. Our findings suggest that size and habitat composition of home ranges influenced coyote use of prey. Specifically, coyote home ranges consisted of a mixture of open and densely vegetated habitats (Fig 4) and it is likely that heterogeneity of habitat types within home ranges provide alternative prey for coyotes [59,61,76–78]. For example, coyote use of small mammals was positively associated with vegetation density and negatively associated with hardwood forest, whereas consumption of rabbits was negatively correlated with vegetation density. Likewise, deer were an important food resource year-round in both study areas, although consumption of white-tailed deer varied considerably among packs. Coyote use of deer was associated with cooler months, small home-range sizes, and less vegetation density. This was not surprising as coyotes exhibit strong selection for open habitats that improve foraging capabilities, such as improved vision and olfaction, and reduced vegetation to allow pursuit of deer and rabbits [7,36,59,61,79,80]. Therefore, we suggest that heterogeneity in habitat composition of home ranges may increase the proportion of available prey to coyotes through spillover of prey populations colonizing sink habitats [81–83]. In other words, spatial heterogeneity within their home ranges allows coyotes to acquire environmental factors (e.g., food, breeding habitat, bed sites) responsible for the distribution of prey, and the continued occurrence of prey within coyote territories is likely supported through dispersal of prey from proximate source habitats [84,85].

Searching for prey involves important costs per unit of time [86–89], and the observed negative correlation between home-range size and use of white-tailed deer suggests that resident coyotes are likely confronted with an important energetic balance. Preying on deer may provide coyotes with greater net energy per unit of search time than smaller prey, and we noted coyote use of rabbits was positively correlated with home-range size. Nevertheless, to maximize net energy gains from diets consisting mostly of small particle-sized food, such as invertebrates and small mammals, coyotes may require larger home ranges to satisfy mass-related energetic requirements, as smaller prey have lower absolute energy than larger prey and are relatively patchy and temporally limited in distribution and availability [90]. Indeed, it is well established that home-range size is inversely correlated with habitat quality [91,92]. Hence, the correlation of relatively larger home ranges and greater use of rabbits may indicate that these resident coyotes were inhabiting lower quality habitat than coyotes with smaller home ranges and greater use of deer.

Previous studies suggested that coyote predation of white-tailed deer in the southeastern United States occurred primarily on fawns during summer [10,93–97], that predation on adult deer was low [98–100], and most consumption of deer during winter was a result of scavenging of carcasses discarded by deer hunters [14,101,102]. Overall, our MMB estimates suggested that the average coyote pack consumed approximately 270 kg of deer per year and annual consumption of deer per pack varied between 55 kg and 490 kg. We observed consistent use of deer throughout the year; adult deer were consumed during all months, and factors influencing deer use differed among adults and fawns. Specifically, use of adult deer was associated with cooler months and smaller home ranges, whereas use of fawns was strongly associated with only warmer months. The strong seasonal influence on use of fawns was not surprising, as fawns were an available prey source only during 8 months of the year and occurred significantly in coyote scats during the height of the fawning season (April—August; Fig 3), when they were most susceptible to coyote predation during their first few weeks of life [10,95,103,104]. However, the observed use of adult deer by coyotes is contrary to previous studies, and year-round use of adult deer suggests that predation plays an important role for coyotes to acquire deer.

Although coyotes will opportunistically consume white-tailed deer killed or wounded by hunters, the availability of carcasses depends on the location of deer mortality [105], and the spatial distribution of hunted lands varies across the landscape, as does hunting pressure and hunting activity [106]. Consequently, coyotes would require large foraging radii to track temporal and spatial variation in carrion availability. Therefore, we believe the negative correlation between coyote home-range size and use of adult deer suggests that coyotes acquired deer through predation. Additionally, despite the widespread distribution of feral pigs across our study areas and the availability of pig carcasses, we rarely observed pig remains in coyote scats, nor did we commonly detect species frequently found as roadkill, further suggesting that scavenging is not an important foraging strategy for resident coyotes in the southeastern United States.

Coyote use of white-tailed deer during the hunting season may relate to seasonal changes in deer movements due to breeding activities, as evidenced by increased deer-vehicle collisions during fall and winter [107–109]. Deer are known to increase movements to exploit mating opportunities and use riskier habitats to avoid human hunters [108–112]. Consequently, changes in deer space use patterns may increase deer presence in areas occupied by coyotes and expose adults and juveniles to greater risk of predation, likely explaining use of deer we observed during peak deer breeding seasons across both study areas. Coyote use of adult deer could be influenced by decreased body condition and rut-related injuries of male deer, making them more susceptible to predation [113]. Likewise, we suspect that predation of juvenile deer accounted for some of the observed use of deer, particularly those that are not members of matriarchal family groups (i.e., males, orphaned females) may suffer greater mortality to predation than adults, as they are solitary individuals encountering seasonal changes in human activity and resource availability for the first time and may be prone to riskier decision-making [114]. Despite studies reported that predation on juveniles was a relevant source of mortality [6,113,115–117], there is a dearth of information detailing mortality and predation rates of juvenile deer, as most studies focus on fawns or deer ≥1.5 years of age. Therefore, we suspect that some adult hair (based on diameter) we recovered from scats belonged to juvenile deer and speculate that perhaps coyote predation on juvenile deer is more common than previously thought.

We noted that differential use of white-tailed deer, rabbits, small mammals, and fruit was influenced by season, and size and habitat composition of coyote home ranges, indicating that resident coyotes can exploit a fluctuating prey base despite constrained space use. For example, it is well known that coyotes consume fruit during summer and some studies suggest that fruit may buffer coyote predation of fawns [7,13,63]. Similarly, we observed seasonal use of fruits, as mean monthly temperature had a strong association with coyote use of fruit. However, we also observed a positive association of fruit consumption with vegetation density. This is not surprising as coyotes exhibit broad use of fruits (i.e., persimmons, blackberry, pears, plums, muscadine, peaches, *Smilax* spp.) that become available during spring through fall and occur in a diversity of habitats. Additionally, our findings suggest that use of fruit was opportunistic as use of mammalian prey did not appreciably decrease with increasing use of fruit. Mean monthly FO of mammalian prey was 78.6% and varied between 60.0–95.8%, whereas mean monthly FO of fruit was 33.8% and ranged between 0.7–71.1%. This pattern suggests that coyotes in the southeastern United States rely primarily on nutritionally superior mammalian prey and supplement their diet with fruit when available, as mammalian prey provide > 3.75 times more energy (KJ/g dry wt) than fruit [118–120].

The presence of coyotes facilitates complex ecological interactions by exerting cascading effects on prey populations, and their predation on white-tailed deer creates conflicts with sustained harvests of deer in regions of the southeastern United States [9–11]. Furthermore, coyotes do not coexist with other large carnivores throughout most of the southeastern United States and contend with lower medium-to-large prey diversity relative to western counterparts [1,16,121]. Consequently, coyotes may exert strong top-down effects on southeastern ecosystems, and recent studies in these ecosystems suggest the presence of coyotes may negatively influence white-tailed deer foraging behaviors and recruitment [7,10,122,123]. Despite these top-down effects on local prey populations, strong site fidelity by resident coyotes, as exhibited by the relative spatial stability of home ranges [36,124], indicates they are defending a finite area while foraging commensurate with the distribution and availability of prey in their territories [73,74]. Because coyotes experience saturation and depletion relationships with their prey [125–127], we offer that differential use of prey by coyotes is influenced by habitat heterogeneity within home ranges, and prey-switching behaviors may stabilize local interactions between coyotes and their prey to permit maintenance of stable territories. Indeed, heterogeneous landscapes and density-dependence are known to stabilize predator-prey interactions through frequency-dependent prey-switching [1,5] and by providing refugia for prey [77,128]. Future assessment of the effects of these factors on distribution and abundance of coyotes is essential to understand effects of coyote predation on mammalian communities in the southeastern United States.

## Supporting information

S1 Dataset Home range size, extent of 7 habitats in home ranges, extent of predicted habitat for 3 prey types in home ranges, vegetation biomass, and principal components scores for coyote territories, Alabama and the Savannah River area (Georgia and South Carolina), 2016. (CSV)

S2 Dataset Frequency of occurrence of prey in coyote scats and environmental covariates used for the generalized linear mixed models, Alabama and the Savannah River area (Georgia and South Carolina), 2016. (CSV)
